# Supplementary material for: Light Pollution Disrupts Seasonal Differences in the Daily Activity and Metabolic Profiles of the Northern House Mosquito, Culex pipiens
Source: Insects. 2023 Jan 10;14(1):64. doi: 10.3390/insects14010064 (PMC9865375; doi:10.3390/insects14010064)
Supplement: Supplementary file 1 [file insects-14-00064-s001.zip › insects-2058730-supplementary.pdf]

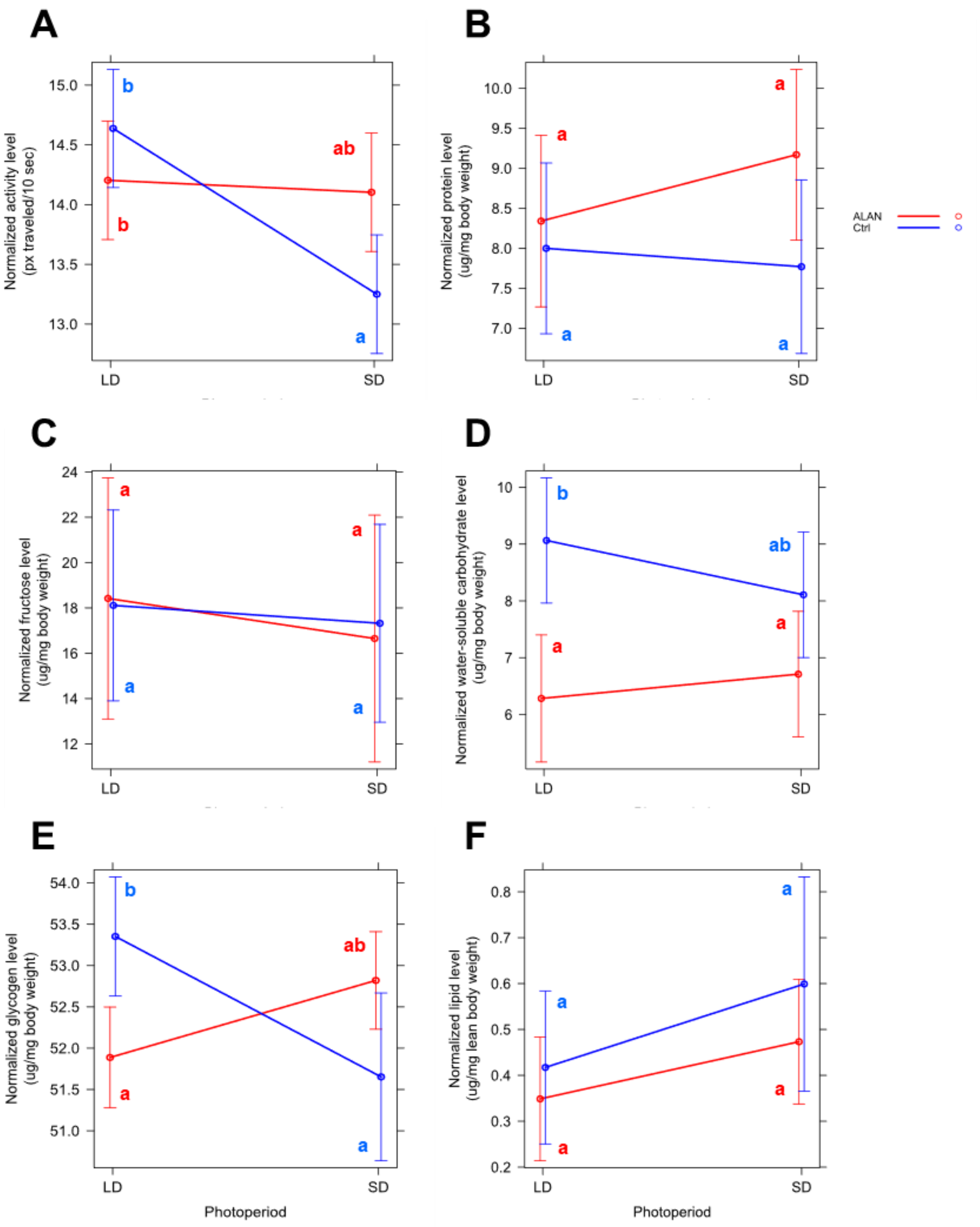

Figure S1. Effects plots showing how photoperiod in the presence (red) and absence (blue) of light pollution influences seasonal differences in mosquito circadian activity and metabolite levels. (A) Activity level in LD mosquitoes was significantly higher than SD mosquitoes in the absence of ALAN, while ALAN resulted in a slight increase in activity under SD conditions which approached the level of significance (Tukey's HSD, $z = -2.393$, $p = 0.078$). Neither protein level (B) nor fructose level (C) varied significantly among mosquitoes in different rearing conditions. (D) ALAN significantly suppressed the accumulation of water-soluble carbohydrate in LD conditions, but not in SD conditions. (E) LD mosquitoes accumulated significantly more glycogen than SD mosquitoes in the absence of ALAN, and ALAN significantly suppressed the accumulation of glycogen in LD conditions. There was no significant difference between LD and SD mosquitoes in the presence of ALAN. (F) Lipid content did not vary significantly among mosquitoes in different rearing conditions. Points indicate means calculated for each normalized dataset; while

vertical bars represent the 95% confidence interval. Letter codes indicate statistically different groups as calculated by Tukey’s Honest Signficant Difference tests.
